# A novel, homozygous nonsense variant of the *CDHR1* gene in a Chinese family causes autosomal recessive retinal dystrophy by NGS‐based genetic diagnosis

**DOI:** 10.1111/jcmm.13841

**Published:** 2018-08-30

**Authors:** Jiewen Fu, Lu Ma, Jingliang Cheng, Lisha Yang, Chunli Wei, Shangyi Fu, Hongbin Lv, Rui Chen, Junjiang Fu

**Affiliations:** ^1^ Key Laboratory of Epigenetics and Oncology The Research Center for Preclinical Medicine Southwest Medical University Luzhou Sichuan China; ^2^ Institute of Medical Technology, Xiangtan Medicine and Health Vocational College Xiangtan Hunan China; ^3^ The Honors College University of Houston Houston Texas; ^4^ Department of Molecular and Human Genetics Baylor College of Medicine Houston Texas; ^5^ Department of Ophthalmology Affiliated Hospital of Southwest Medical University Luzhou Sichuan China

**Keywords:** *CDHR1* gene, expression, nonsense mutation, retinal dystrophy, targeted next‐generation sequencing

## Abstract

Retinal dystrophy is an inherited, heterogeneous, chronic and progressive disorder of visual functions. The mutations of patients with autosomal recessive retinal retinopathy cone‐and‐rod dysfunction and macular dystrophy have not been well described in the Chinese population. In this study, a three‐generation Chinese retinal dystrophy family was recruited. Ophthalmic examinations were performed. Targeted next generation sequencing (TGS) was used to identify causative genes, and Sanger sequencing was conducted to verify candidate mutations and co‐segregation. Reverse transcription (RT)‐PCR was applied to investigate the spatial and temporal expression patterns of *cdhr1* gene in mouse. A novel, homozygous, deleterious and nonsense variant (c.T1641A; p.Y547*) in the *CDHR1* gene was identified in the family with autosomal recessive retinal dystrophy, which was co‐segregated with the clinical phenotypes in this family. RT‐PCR analysis revealed that *cdhr1* is ubiquitously expressed in eye, particularly very high expression in retina; high expression in lens, sclera, and cornea; and high expression in brain. In conclusion, our study is the first to indicate that the novel homozygous variant c.T1641A (p.Y547*) in the *CHDR1* gene might be the disease‐causing mutation for retinal dystrophy in our patient, extending its mutation spectrums. These findings further the understanding of the molecular pathogenesis of this disease and provide new insights for diagnosis as well as new implications for genetic counselling.

## 1 INTRODUCTION

Retinitis pigmentosa (RP) is a large, genetically heterogeneous group of inherited ocular diseases that results in a progressive retinal degeneration.^1^, ^2^, ^3^ Inheritance patterns in RP include autosomal recessive (arRP), autosomal dominant (adRP) and X‐linked inheritance (xlRP). Patients with a retinal dystrophy of autosomal recessive pattern present symptoms of cone‐and‐rod dysfunction and macular atrophy.^4^ Cone‐rod dystrophy (CRD), or retinal dystrophy, is either syndromic or non‐syndromic RP (mostly non‐syndromic RP) with autosomal dominant, autosomal recessive or X‐linked recessive inheritance, presenting early loss of cone photoreceptors and a parallel or subsequent loss of rod photoreceptors. The loss of cone photoreceptor cells leads to visual loss, visual field loss, abnormalities of colour vision and variable degrees of photophobia and nystagmus, whereas the loss of rod function leads to night blindness.^5^ In many cases, successive generations (50%~60%) are inherited as autosomal recessive, resulting from homozygous mutations in the RP‐related genes. Mutations in the *CDHR1* gene (OMIM 609502) lead to autosomal recessive retinal dystrophy or autosomal recessive CRD (OMIM 613660).^5^, ^6^, ^7^

The *CDHR1* (Cadherin‐Related Family Member 1), also called *PCDH21* (Protocadherin‐21), *CORD15*,* PRCAD* or *RP65*, is located at chromosome 10q23.1; CDHR1 belongs to cadherin repeat domain‐containing protein. Cadherin repeat domain‐containing protein is very similar to the cadherins,^8^ which are calcium‐dependent cell adhesion proteins that preferentially interact with themselves in connecting cells, and calsyntenins, which modulate calcium‐mediated postsynaptic signals. CDHR1 is a photoreceptor‐specific cadherin,^9^ and immunoprecipitation studies showed that CDHR1 are also found in complexes with PROM1 and actin filaments, playing a critical role in photoreceptor disk morphogenesis.^10^

Here, we applied targeted next‐generation sequencing (TGS) technology, the most available ‎and promising method available,^11^, ^12^, ^13^, ^14^, ^15^, ^16^ to identify a novel, homologous mutation of *CDHR1* gene in a Chinese family with autosomal recessive retinal dystrophy, extending the gene's mutation spectrum.

## 2 MATERIALS AND METHODS

### 2.1 Ethical statement, proband and clinical assessment

**Figure 1:**
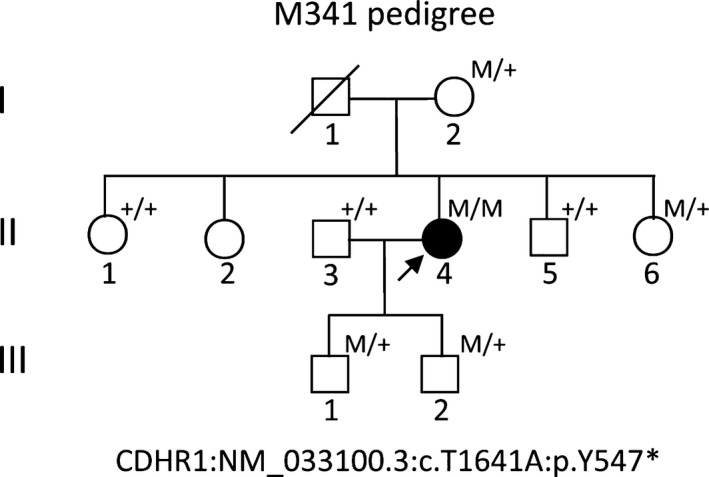
M314 pedigree with retinal dystrophy in the proband. Family number and disease‐causing mutation are presented. Normal individuals are shown as clear circles (females) and squares (males), the affected individual is shown as filled symbol. The patient above the arrow indicates as a proband (II: 4), with the homozygous, nonsense variant of the *CDHR1* gene: NM_033100.3:c.T1641A:p.Y547*, where the asterisk indicates the stop codon. “M” indicates the mutant allele of *CDHR1* (mutant type), whereas “+” indicates the normal allele of *CDHR1* (wild type)

The research was approved by the Ethical Committees of the *Southwest Medical University*; written informed consent conforming to the tenets of the Declaration of Helsinki (1983 Revision) was obtained from all participants.^17^ The study consisted of a proband (Figure 1, pedigree II: 4, arrow), and 9‐related family members from three‐generations, with no consanguineous marriage history, based on their genetic and pedigree analysis (Figure 1). For clinical diagnosis, a detailed clinical history and ophthalmic examinations were performed in proband, including the best‐corrected Snellen visual acuity, humphrey visual fields, ‎slit‐lamp biomicroscopy, fundoscopy, ‎optical coherence tomography, fundus photographs (FP) and fundus fluorenscent photographs (FFP), and standard electroretinography, which were used in previously studies.^17^, ^18^, ^19^

### 2.2 Blood sampling and DNA extraction

Two millilitres of fresh peripheral bloods were taken, and human genomic DNAs (gDNAs) were extracted using the previously described standard phenol/chloroform method from blood leucocytes of the proband and pedigree members who were accessible.^20^, ^21^ Blood samples were also taken from 100 RP‐unrelated, ethnically matched and healthy control volunteers no any disease history.

### 2.3 Capture panel designing, exome sequencing

To access the disease‐causing genes and mutations, the panels for TES analyses on the DNA sample from the proband M341 were designed, according to the Illumina paired‐end libraries (Illumina, Inc., San Diego, CA, USA).^11^, ^12^, ^14^ The capture Agilent probes were used in previously published studies.^11^, ^12^, ^14^, ^18^, ^22^ Two micrograms of extracted proband gDNA was randomly sonicated into 300~500 bp fragments. The 5′ ends of DNA fragments were phosphorylated by polynucleotide kinase, and adenine was added at the 3′ ends. Then hybridization to the pre‐capture libraries was quantified (the PicoGreen fluorescence assay kit, Invitrogen, Carlsbad, CA, USA). Each captured DNA libraries were applied for sequencing on Illumina HiSeq 2000 (Illumina, Inc.) at the BCM core facility, following the manufacturer's protocols, after pre‐capture libraries pooled, washed and recovered (Agilent Technologies, Santa Clara, CA, USA).^18^ Then, paired‐end sequencing illumina reads were aligned to the human hg19 reference genome using Burrows‐Wheeler Aligner version 0.6.1 and available public online UCSC database (http://genome.ucsc.edu/).^23^ Single nucleotide polymorphisms (SNPs) and Insertions/Deletions (INDELs) variations were refined using a toolkit Atlas‐SNP2 and Atlas‐Indel2 (GATK version 1.0.5974).^24^ Variant frequency data were applied to online control databases, CHARGE consortium,^25^ 1000 Genome Project,^26^ ANNOVAR,^27^ ESP‐6500^28^ and Exome Aggregation Consortium (ExAC) databases, to look for the pathogenic mutations in all candidate genes with a minor allele frequency of more than 5%. Sequencing depth 4, estimated copy number 2, SNP quality 20 (score 20 represents 99% accuracy of a base call), and a distance between two SNPs > 5 are considered the filtration criteria for candidate SNPs, according to previously reported studies.^17^, ^18^ Sequence variants should not annotated in any of the above public databases. Finally, we identified the variant in the affected subject (Figure 1, pedigree II: 4).

### 2.4 Primer design, PCR amplification and Sanger sequencing

For mutation verification and co‐segregation analysis, polymerase chain reaction (PCR) amplification and direct Sanger sequencing of variant was applied to gDNA of all the available individuals.^18^ Locus‐specific primer pair (CDHR1‐1641) was designed from the online Primer3 program (http://primer3.ut.ee/) by genomic DNA sequences containing identified mutation c.T1641A in *CDHR1* (Table 1). A product with 208 bps was amplified using gDNA as the template. Then, the PCR products were sequenced by Sanger method on an ABI‐3500DX sequencer (Applied Biosystems Inc., Foster City, CA, USA) through the specific primer CDHR1‐1641L in Table 1. All unrelated ethnical‐matched controls were sequenced using aforementioned primers.

**Table 1:** The sequences of polymerase chain reaction (PCR) primers and PCR product sizes

Primer name	Left primer	Sequence (5′‐3′)	Right primer	Sequence (5′‐3′)	Size	°C
CDHR1‐1641	CDHR1‐1641L	ctgatccacccatccactg	CDHR1‐1641R	tccctagcaccatcgtcttc	208	60
RT‐cdhr1	RT‐cdhr1‐L	gttccctctgctctcatcca	RT‐cdhr1‐R	tccagctcttccaccagagt	383	60
RT‐b‐actin‐m	RT‐b‐actin‐mL	tgttaccaactgggacgaca	RT‐b‐actin‐mL	tctcagctgtggtggtgaag	392	60

### 2.5 Protein structure and bioinformatic analysis

The functional classification of proteins via subfamily domain architectures for CDHR1 was performed through an online NCBI system (https://www.ncbi.nlm.nih.gov/Structure/cdd/wrpsb.cgi).^19^, ^29^, ^30^ Comparison of CDHR1 in different species was also performed by previously online NCBI system.

### 2.6 RNA extraction and revere transcriptional‐polymerase chain reaction (RT‐PCR)

RNAs from mice with indicated ages and indicated tissues were extracted using RNAsimple Total RNA kit according to our previously reported standard protocols,^31^ and the quality was measured by a NanoDrop‐2000‐spectrophotometer (NanoDrop 2000, Wilmington, DC, USA) and an agarose gel electrophoresis. Whole eye balls from embryos at 12.5 days (12 days) and 20.5 days (20 days) before birth for mice were taken instead of retina because of sampling difficulty.

Then, the total amount of RNA in each reaction system is equal to 500 ng for cDNA synthesis (reverse transcriptase/RT) using random oligomer primers method and RT kit. The primer sequences, product size and PCR conditions (anneal temperature) are listed in Table 1. Semi‐quantitative RT‐PCR with a 383 bps product was performed by primer pair RT‐cdhr1 for mouse *cdhr1* gene; *ß‐actin* gene of mouse was served as an internal control by primer pair RT‐b‐actin‐m (Table 1). Each assay was performed in three independent tests.

## 3 RESULTS

### 3.1 Pedigree and clinical characteristics

**Figure 2:**
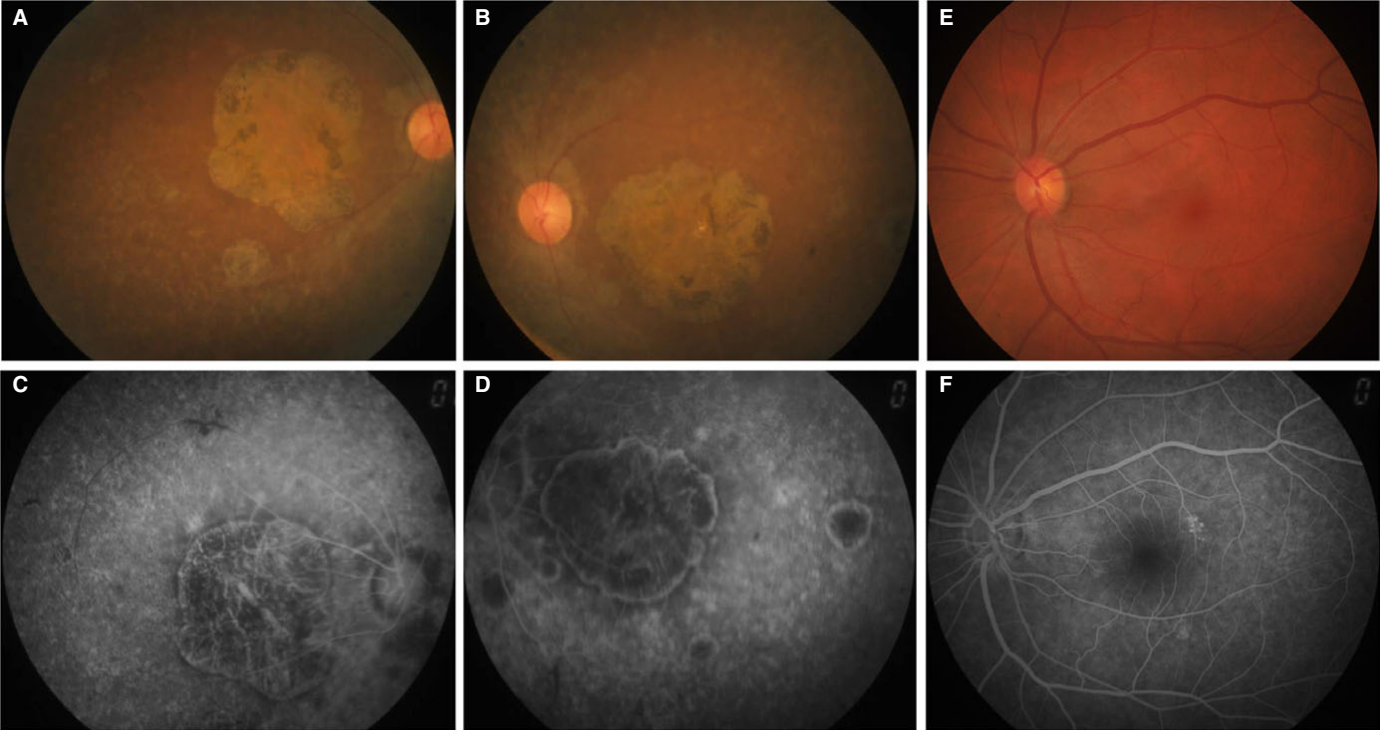
Representative fundus photographs (FP) of patient II:4 from both eyes. A&B, Fundus photographs (FP) of proband (right and left, respectively). C&D, Fundus fluorenscent photographs (FFP) of proband (right and left, respectively). E&F, FP and FFP in a normal control

**Figure 3:**
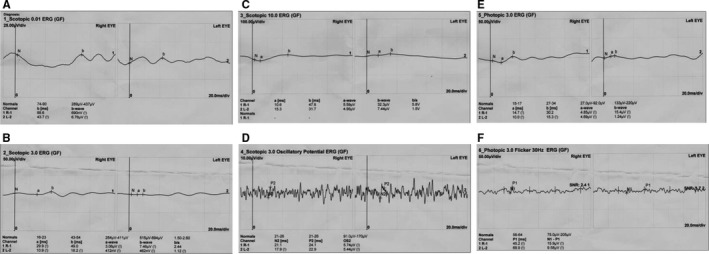
Electroretinography (ERG) in proband II:4 with different waves. The scotopic electroretinograms (ERGs) at 0.01 (A), 3.0 (B), 10.0 (C), scotopic 3.0 oscillatory potential ERG (D), photopic ERG 3.0 (E), and photopic ERG 3.0 flicker ERG 30 Hz (F)

The proband (Figure 1, II: 4) from non‐consanguineous RP family is a 45‐year‐old Chinese female with a clinical signs of progression of blindness characteristic of retinitis pigmentosa; she who noticed the simultaneous onset of dark adaptation difficulties, trouble with colour vision and light sensitivity by age of 24. She did not complain of nyctalopia. She was diagnosed with “optic atrophy” at local hospital at age of 27. In the fourth decade of life, visual acuity was markedly decreased, and colour vision was severely impaired. The family included 10 members and 3 generations, and all others were normal. The proband had no known family history of retinal disease through three generations, making her an isolated case and suggesting an autosomal recessive inheritance manner. The fundus photographs (FP) and fundus fluorescent photographs (FFP) of the proband II: 4 in both eyes are shown in the Figure 2. The image of FP in proband displayed attenuated vessels, absence of the foveal reflex, punctated salt‐ and pepper‐like appearance, circular patches of RPE atrophy both at the macula and in the periphery with associated peripheral pigment migration (Figure 2A~B); further FFP results showed a hyperautofluorescent ring surrounding a central area of hypoautofluorescence and an atrophic macular region (Figure 2C~D). For comparing, Figure 2E~F shows age‐matched normal control in Chinese for FP and FFP. Her electroretinogram (ERG) in different waves showed markedly reduced rod‐and‐cone responses (Figure 3A~F). As a result, the proband in our study was presented with typical retinal dystrophy or macular/cone–rod dystrophy (MD/CRD). Invariably, this proband patient was developmentally normal upon review at the age of 45 years old.

### 3.2 Next‐generation sequencing analysis and putative pathogenic mutation screening

To access RP disease‐causing gene mutation, targeted capture high‐throughput ‎sequencing of 195 RP‐related genes was performed successfully using a capture panel on the gDNA sample of proband (Figure 1, pedigree II: 4). Targeted regions with evenness scores more than 0.8 across of sample were converged. Commonly, 96.0% of the targeted regions have coverage of >20× and 91.1% of the targeted regions have coverage of >40×.^18^ After quality assessment, more than 97% of the billions of bases were aligned to the human reference sequences and, among those, billions of bases were recovered with a 10‐fold coverage target region. Then, causative mutations were identified by automatic variant calling, filtering and annotation pipeline in the capture sequencing data.^12^, ^14^, ^32^ SIFT, Polyphen 2, LRT, MutationTaster, MutationAssessor and dbNSFP were used to filter out non‐pathogenic population variations, which were not annotated in any of the above public databases and were prioritized for further confirmation and characterization. Surprisingly, a single nucleotide homozygous, nonsense variant (c.T1641A) of exon 15 in the *CDHR1* gene (NM_033100.3) in this patient was identified, leading to an amino acid change from Tyrosine (Tyr, Y) to stop codon at position 547 of the CDHR1 protein (p.Y547*), and caused a loss of more than one‐thirds of its C‐terminus (CDHR1: NP_149091.1) (Figure 1 II: 4). The deleterious and pathogenic aspect of c.T1641A (p.Y547*) mutation in the *CDHR1* gene is presented in Table 2. This nonsense variant c.T1641A (p.Y547*) in *CDHR1* gene most likely damaged protein function in the analysis of this Chinese non‐consanguineous RP family. This variant was searched in the ExAC and HGMD databases and found as a novel mutation (Table 2). Other variants in genes for *CROCCP2*,* SLC6A6*,* RP1*,* MYO7A*,* RDH5*,* FBLN5*,* RLTPR* and *GPR179* by NGS were excluded as deleterious and pathogenic mutations.

**Table 2:** Characteristics of CDHR1 variant in a retinal dystrophy patient

Gene	Exon	Variation				ExAC
		Nucleotide	Protein	Type	Status	
CDHR1	15	c.T1641A	p.Y547^a^	Nonsense	Homo	Novel

a Stop codon; c, variation at cDNA level; CDHR1, cadherin‐related family member 1; Homo, homozygote; p, variation at protein level.

### 3.3 Mutation verification and segregation analysis

**Figure 4:**
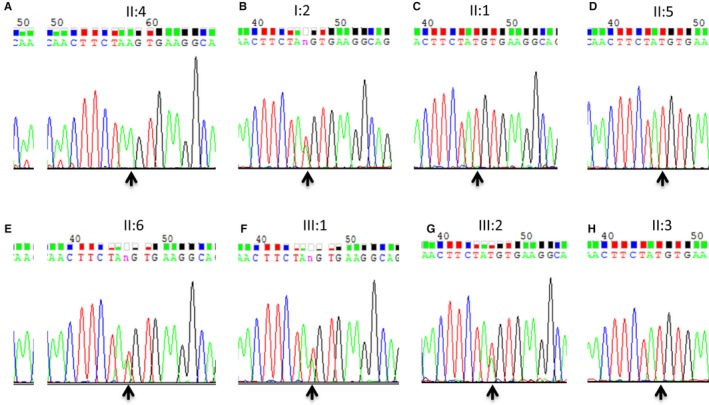
Photogram profiles for validation by Sanger sequencing. A~H, indicate the sequencing results in II: 4 (mutant homozygous type), I: 2 (heterozygous type), II:1 (wild type), II:5 (wild type), II: 6 (heterozygous type), III: 1 (heterozygous type), III: 2 (heterozygous type), and II:3 (wild type, normal male from no eye disease history family), respectively. The arrows indicate mutation at the nucleotide position NM_033100.3:c.T1641A in the *CDHR1* gene

Albeit deficient, the Sanger sequencing was used for confirmation and segregation analysis (Figure 4). The c. T1641A variant of *CDHR1* was confirmed in the mutant homozygous type patient (pedigree II: 4; Figure 4A), and identified mutant heterozygous types in proband mother as a carrier (pedigree I: 2; Figure 4B), wild types with normal phenotype of proband's elder sister and younger brother (pedigree II: 1, II: 5; Figure 4C,D,), mutant heterozygous type with normal phenotype of proband's younger sister (pedigree II: 6; Figure 4E), and mutant heterozygous types with normal phenotype of proband's two sons (pedigree III: 1, III: 2; Figure 4F&G) in the family; the husband of proband with wild type showed no mutation (pedigree II: 3; Figure 4H). Thus, the c. T1641A variant of *CDHR1* was co‐segregated with the disease phenotype in all the family's members we tested. This homozygous mutant was absent in 100 unrelated, normal, ethnically matched controls (data not shown). Notably, proband's father I: 1 with normal phenotype was not available because of death, and another proband's elder sister II: 2 was not available either. But this father may carry the same variant c. T1641A by pedigree analysis (pedigree I: 1; Figure 1).

All together, these findings show complete co‐segregation in the pedigree for the retinal dystrophy family and pinpoint its role in MD/CRD pathogenesis.

### 3.4 Functional effects of pathogenic variant c.T1641A (p.Y547*) for CDHR1 and *cdhr1* mRNA expression profiles

**Figure 5:**
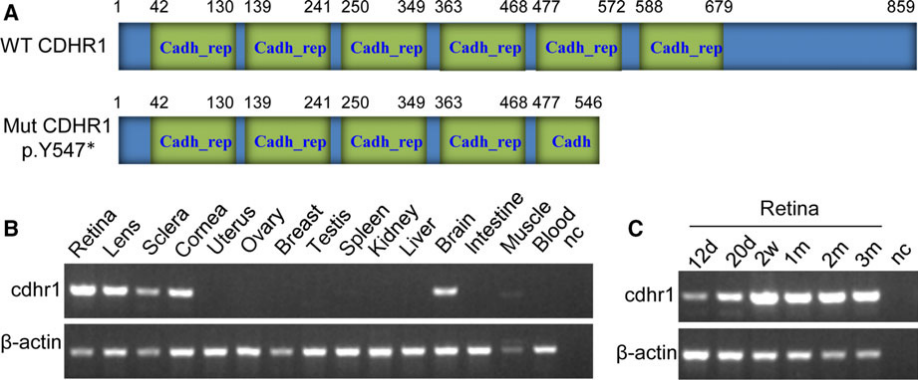
CDHR1 protein structure and *cdhr1* gene expression. A, CDHR1 structure and position for variant p.Y547*, which is corresponding to the gene of *CDHR1*: NM_033100.3:c.T1641A. Expressions for *cdhr1* mRNA in different tissues (B) and in different development stages or times of the retinal tissue (C) in mice. Cadh_rep, cadherin repeat; WT, wild type; Mut, mutant type; d, day(s); w, week(s), m, month(s); nc, negative control without any template cDNA; muscle, skeletal muscle. Whole eye balls at 12.5 d (12 d) and 20.5 d (20 d) from embryos in panel C, respectively

CDHR1 structure and position for variant p.Y547* are shown in Figure 5. Searching through the Conserved Domain Database (CDD) in NCBI revealed that CDHR1 has six cadherin repeat domains (Figure 5A), which are calcium‐dependent cell adhesion proteins that preferentially interact with themselves in connecting cells, and calsyntenins, which modulate calcium‐mediated postsynaptic signals. Mutation of p.Y547*, located within fifth cadherin repeat domain, leads to a loss of more than one‐thirds of CDHR1 at the C‐terminus, including almost one and half of cadherin repeat domains (Figure 5A), possibly changing its function. Comprehensively, this study shows that recessive *CDHR1* homozygous mutations, c.T1641A (p. Y547*), which most likely causes ‎disease of retinal dystrophy.‎

Expressions for *cdhr1* mRNA in 15 different tissues and 6 different development stages of retina were investigated from mice. The results showed that *cdhr1* transcript is very highly expressed in retina, lens, sclera, cornea of eyes and brain; very weakly expressed in skeletal muscle; had no detectable expressions in other nine tissues tested (Figure 5B); and highly expressed in the 6 different development stages/times of retinal tissue (Figure 5C). Very high expression in the retina and ubiquitous expression in different tissues from eye indicated that CDHR1 plays important roles in retina/eye functions.

## 4 DISCUSSION

In this study, we identified a homozygous, nonsense variant c.T1641A (p.Y547*) of the *CDHR1* gene in a non‐consanguineous marriage Chinese family, which produced a truncated protein and lead to a loss of more than one‐thirds of CDHR1 at the C‐terminus, including almost one and half of cadherin repeat domains, and possibly changed its function of protein–protein interactions causing to retinal dystrophy disease. By searching the Human Gene Mutation Database (http://www.hgmd.cf.ac.uk/ac/gene.php?gene=CDHR1) (access date, July 4, 2018), only 15 pathogenic variants have been reported, including missense/nonsense (8), splicing (3), small deletions (3) and small insertion (1). To the best of our knowledge, *CDHR1* variant c.T1641A (p.Y547*) is a novel mutation, extending its mutation spectrums. Thus, this finding shows that the recessive *CDHR1* mutations, c.T1641A (p. Y547*), likely cause ‎disease of retinal dystrophy in our studied Chinese pedigree.

The pathogenic *CDHR1* mutation was first identified in 2010,^5^, ^7^ and only a few mutations have been reported since then.^4^, ^33^, ^34^, ^35^, ^36^, ^37^ Very recently, six *CDHR1* mutations were also identified in Germany for macular and cone/cone‐rod dystrophies or retinal dystrophy.^16^, ^38^ These small amounts of mutations suggest that the mutations in the *CDHR1* gene are a rare case of arCRD (autosomal recessive cone‐rod dystrophy) in Western countries.^4^, ^36^ We identified a homozygous variant c.T1641A of the *CDHR1* gene from a non‐consanguineous marriage Chinese family, indicating that the incidence of this variant in Chinese population may be higher. Further studies for genetic epidemiology or allele frequency should be conducted in the Chinese population.

In literatures, different autosomal recessive phenotypes have been associated with the *CDHR1* gene mutations, ranging from RP to CRD although the relationship between phenotypes and gene mutations are variable.^16^, ^33^, ^34^, ^36^, ^38^ The proband in our study, with a loss of more than one‐thirds of CDHR1 by a homozygous and nonsense variant p.Y547*, has been noticed the simultaneous onset of dark adaptation difficulties, trouble with color vision, and light sensitivity at age of 24; ERG in different waves showed markedly reduced rod‐and‐cone responses; FP/FPP showed macular dysdrophy (MD). Clinically, this patient presented MD/CRD. Thus, combined with our study, patients with CDHR1 truncated protein by nonsense, splicing variants or deletions with frameshift, other than missense variants, might cause more severely phenotypes, such as MD/CRD, and/or earlier disease onset.^16^, ^33^, ^34^, ^36^, ^38^, ^39^

Alternatively spliced transcript variants of *CDHR1* encode two different isoforms; isoform 1 (NP_149091.1) has 859 amino acids, whereas isoform 2 (NP_001165442.1) has 745 amino acids; both are identical at the first 680 amino acids containing 6 cadherin repeat domains. Variant p. Y547* in both isoforms of CDHR1 usually lose one and half of cadherin repeat domains, likely causing disease. *CDHR1* is highly expressed in the retina, more specifically in the junction between the inner and outer segments (OS) of rod and cone photoreceptors.^9^ Our study using the mouse model shown that *cdhr1* mRNA levels are very highly expressed in retina; highly expressed in lens, sclera, cornea and brain; and weakly expressed in skeletal muscle in 15 different tissues. High expression in the 6 different development stages/times of retina was also shown. Higher expression in brain is consistent with the report made by Nakajima et al,^8^ but not the report made by Rattner et al.^9^ But no matter what, higher expression in retina and ubiquitous expressions in different tissues of eye indicated that CDHR1 plays an important role in retina/eye functions.

In conclusion, our study was the first to identify that the homozygous variant c.T1641A (p.Y547*) of the *CHDR1* gene is most likely the disease‐causing mutation for retinal dystrophy in our Chinese patient, extending its mutation spectrums. Targeted next‐generation sequencing (TGS) technology provides us an accurate, rapid and cost‐effective molecular method for gene diagnosis. These findings facilitate better understanding of the molecular pathogenesis of the disease, provide new insights for diagnosis and have implications for genetic counselling.

## CONFLICT OF INTEREST

The authors declare no conflict of interest.

## AUTHOR CONTRIBUTION

J.F. was in charge of idea, project design and concept of the manuscript. J.F., L.M., J.C. and C.W. performed PCR amplification, Sanger sequencing and data analysis. S. F. and R.C. did experiment of NGS and analysed data. J.C. and J. F. performed DNA extraction. H. L. recruited the clinical patient and in charge of clinical assessment. J. F. and S.F. wrote and revised the manuscript.

## PATIENT CONSENT

Obtained.

## ETHICS APPROVAL

The study has the Ethical Committees approval granted by the *Southwest Medical University*.
